# Assessment of outcomes of elective cancer surgeries in children during coronavirus disease 2019 pandemic: Retrospective cohort study from a tertiary cancer center in India

**DOI:** 10.1097/MD.0000000000026752

**Published:** 2021-09-03

**Authors:** Sajid S. Qureshi, Deepak Ramraj, Girish Chinnaswamy, Badira C. Parambil, Maya Prasad, Nayana Amin, Subramaniam Ramanathan, Nehal Khanna, Siddharth Laskar

**Affiliations:** a Division of Paediatric Surgical Oncology, Department of Surgical Oncology, Tata Memorial Hospital and Advanced Centre for Training Research and Education in Cancer (ACTREC), Tata Memorial Centre, Mumbai, India; b Homi Bhabha National Institute (HBNI), Mumbai, India; c Division of Pediatric Oncology, Department of Medical Oncology, Tata Memorial Hospital and Advanced Centre for Training Research and Education in Cancer (ACTREC), Tata Memorial Centre, Mumbai, India; d Department of Anaesthesia, Tata Memorial Hospital, and Advanced Centre for Training Research and Education in Cancer (ACTREC), Tata Memorial Centre, Mumbai, India; e Department of Radiation Oncology, Tata Memorial Hospital and Advanced Centre for Training Research and Education in Cancer (ACTREC), Tata Memorial Centre, Mumbai, India.

**Keywords:** coronavirus disease 2019, elective cancer surgeries, pediatric cancers

## Abstract

To describe the outcomes of elective cancer surgeries and adverse consequences on the patients and medical staff due to the surgical interventions in children during the Coronavirus Disease 2019 (COVID-19) pandemic.

The study included children younger than 15 years who underwent elective cancer surgeries from March 4, 2020 and December 3, 2020.

A total of 121 patients (62% male; median age, 3 years) underwent surgery. The surgical procedures included nephrectomies (n = 18), neuroblastoma (n = 26) and soft tissue tumor resections (n = 24) and complex surgical procedures like extended liver resections (n = 2), intra-atrial thrombectomy under cardiopulmonary bypass (n = 2), pancreatoduodenectomy (n = 1), and free microvascular flaps (n = 7). Clavien-Dindo Grade III complications were 5% (n = 6), and there were no postoperative deaths. Preoperative COVID-19 testing was performed in 82% of children, and only 2% showed severe acute respiratory syndrome coronavirus 2 positivity. Postoperatively, 26 children were tested because of specific symptoms and, 6 tested positive for severe acute respiratory syndrome coronavirus 2. Except for a median delay of 23 days in treatment, none of the patients with COVID-19 required critical hospital management. None of the surgical residents or faculty acquired COVID-19, while 4 each medical and support staff were tested positive in the study period.

COVID-19 was not a deterrent for continued cancer care, and surgeries could be safely performed adopting universal preventive measures without any added morbidity from COVID-19. Caregivers and centers dealing with childhood cancers can be encouraged to sustain or seek early healthcare.

## 1 Introduction

The World Health Organization declared coronavirus disease 2019 (COVID-19) a pandemic in February 2020 after a widespread outbreak of severe acute respiratory syndrome coronavirus 2 (SARS-CoV-2). A near-complete lockdown was imposed in several countries, including India, since March 2020, and Mumbai being an epicenter, was severely affected by the pandemic and the lockdown. Stringent travel restrictions and diversion of resources produced a sudden disruption in the diagnosis, planning, and treatment of patients. Tata Memorial Hospital (TMH), Mumbai is the largest comprehensive cancer center in India, providing treatment and opinions to over 75000 new patients annually.^[2]^ Unlike other elective treatments, cancer therapies cannot be stopped even in a pandemic given devastating consequences to the patient. To ensure continuity of appropriate curative and palliative treatment effectively and simultaneously protect the health and safety of the medical, nursing, and support staff, TMH initiated several preventive measures well in advance of the lockdown.^[1,2]^

Pediatric oncology revolves around the tenet of timely detection, diagnosis, and coordinated systemic and local therapy delivery. Any delay or disruption of the treatment protocol can jeopardize cure rates. The Department of Surgical Oncology at TMH continued elective cancer surgeries, albeit de-escalated by approximately 50% during the initial period of the COVID-19 pandemic.^[3]^ In tandem, the Pediatric Surgical Oncology division also sustained surgical services, including diagnostic, emergency, and elective procedures for children. The service slowly scaled up its efforts with the increasing availability of operating rooms (OR) and rising confidence of OR personnel with specialized protection and stringent standard operating procedures (SOP).

Rising to the challenge, the international childhood cancer community provided practical advice for the management of cancers during the pandemic, which included therapy adaptation for low and middle-income countries.^[4]^ Preliminary reports also suggested that pediatric cancer patients may not be more vulnerable than other children and anticancer treatment can continue with no significant adjustments.^[5–7]^ However, data on pediatric cancer treatment, especially the surgical management and outcomes during the pandemic, is scarcely reported. Therefore, we analyzed the outcomes of the elective cancer surgeries and adverse consequences on the patients and medical staff due to the surgical interventions in a COVID hotspot. In addition, delays in surgical or adjuvant treatment due to COVID-19 and the shortfall in operative procedures and new registrations compared to the corresponding period during the previous year were also surveyed.

## 2 Methods

The Institutional Review Board approved the study, and a waiver of consent was taken. The study included children younger than 15 years; this age was selected based on the institutional policy for the pediatric age group. The inclusion criteria were a histological/cytological confirmed diagnosis of malignancy and elective cancer surgeries performed from March 4, 2020 to December 3, 2020. Data were retrieved from a prospectively maintained database. Preoperative COVID-19 test became mandatory from April 18, 2020 at our institute. Prior to this, patients were selected for surgery based on the absence of symptoms (suggestive of COVID-19) and no prior contact or relevant travel history. Caregivers accompanying patients for admission were tested only in the presence of symptoms or provided a history of high-risk contact in the preceding 14 days. The test was performed on nasopharyngeal and oropharyngeal swabs by TaqMan probe-based real-time reverse transcriptase-polymerase chain reaction (RT-PCR) method. Patients who tested positive and asymptomatic/mildly symptomatic were isolated at home. At the same time, those with symptoms were admitted to makeshift wards for supportive care or particular earmarked areas for intensive care in the hospital. The infected patients were operated on when they had 2 consecutive negative results of the RT-PCR test (one week apart starting the 14th day after the initial positive test). For major surgeries, where blood loss or postoperative intensive care was anticipated, meticulous planning and coordination with the blood bank and the intensive-care unit (ICU) for the availability of blood or blood products and ICU beds were enforced. For emergency surgeries, where there was limited time to wait for an RT-PCR result or if the patient had COVID-19, a dedicated COVID-19 OR with appropriate SOPs was established. All treatment decisions were made at the multidisciplinary tumor board meeting via videoconferencing. The details of the COVID-19 preventive measures adopted at TMH are previously described and were strictly adhered to.^[1,2]^ The SOP regarding personal protection equipment (PPE) in OR included an N95 mask and a face shield, a surgical gown with a plastic apron, head cap, and gloves. Proper disposal of PPE was practiced. The number of personnel was restricted in the OR at all times. When aerosol-generating procedures were performed like endotracheal intubation, tracheostomy, bone cutting or drilling, etc., the non-essential staff was sent out of the OR. Postoperative complications were defined according to the Clavien–Dindo (CD) classification.^[8]^ CD Grade I and II were considered minor, and Grade III and above were considered major complications. Severe COVID-19 illness is defined as a diagnosis of pneumonia, respiratory distress, or failure, or sepsis. Demographic data, RT-PCR test results, operative procedures, complications, if any, and delay in treatment were recorded. We compared the number of new cancer case registrations and surgeries performed during this period to the corresponding period of the previous year (March 2019 to November 2019) to determine the change in the patient population.

Data were recorded in the IBM SPSS platform and analyzed using the SPSS version 25. Descriptive analysis was performed to identify the distribution of variables. For the various continuous variables, means, medians, and an interquartile range were calculated, and for the categorical variables, proportions were noted.

## 3 Results

During the study period, 121 elective surgeries were performed. All elective surgeries were performed in the allotted OR without unwarranted rescheduling for any patient. Minimally invasive surgeries were not performed on any patients. Demographic and operative outcomes are summarized in Table 1. The youngest child was 4 months old, and 67% were under 4 years of age. Abdominal and pelvic surgeries constituted 55.4% of all procedures, followed by head and neck (23%) and thoracic (10.7%) surgeries. The list of surgical procedures for various tumor types is presented in Table 2. Plastic reconstructive procedures were required in 17% of the cases, which included free microvascular flaps (n = 7), local fasciocutaneous flaps (n = 6), a prosthetic implant for mandibular reconstruction (n = 2), vascular graft (n = 1), nerve graft (n = 2), and, skin graft (n = 2). Postoperative CD grade III complications occurred in 6 (5%) patients, and there was no mortality. In addition to the elective surgeries, 4 emergency surgeries and 85 minor procedures were performed during this period which included chemoport insertions (25), chemoport removals (24), and biopsies from various sites (25), and other miscellaneous procedures.

**Table 1:** Patient characteristics and perioperative details.

Characteristics	No. (%)
Age, median (IQR^∗^) yr	3 (1–6)
Age group yr	
<1	9 (7.4)
1–4	72 (59.5)
5–10	22 (18.2)
11–15	18 (14.9)
Gender	
Male	75 (62)
Female	46 (38)
Region	
Head and Neck	28 (23.1)
Thorax	13 (10.7)
Abdomen and Pelvis	67 (55.4)
Soft Tissue	11 (9.1)
Thoraco-abdominal	2 (1.7)
Complications	
Overall	26 (20.8)
Minor (CD^∗∗^ Grade I and II)	18 (14.8)
Major(CD Grade III)	6 (5)
Re-explorations	
Yes	1
No	120
Mortality	Nil

^∗^IQR = interquartile range, ^∗^CD = Clavien Dindo score.

**Table 2:** Tumor types and surgical procedures performed.

Tumor type	No. (%)
Neuroblastoma	26 (21.5)
Abdominal	21
Mediastinal	3
Cervicothoracic	1
Pelvis	1
Soft tissue tumors	24 (19.8)
Rhabdomyosarcoma	11
Non-rhabdomyosarcoma	13
Renal tumors	18 (14.9)
Unilateral nephrectomy	11
Bilateral partial nephrectomy	4
Nephrectomy with intra-atrial thrombectomy	2
Nephrectomy with IVC thrombectomy	1
Liver tumors	13 (10.7)
Partial hepatectomy	10
Extended hepatectomy	2
Hepatectomy with Whipple's procedure	1
Jaw tumors	9 (7.4)
Maxillectomy	5
Mandibulectomy	2
Combined	2
Others	31 (25.7)
Germ cell tumors	7
Retinoblastoma enucleations	6
Chest wall Ewing's sarcoma	4
Salivary gland tumors	3
Miscellaneous	11

Preoperative COVID-19 test was not performed in 22 (18%) patients as they were operated on before April 18, 2020. None of these patients developed any COVID-related symptoms in the postoperative period. Of the 99 preoperatively tested asymptomatic patients, only 2 (2%) showed SARS-CoV-2 positivity. The surgery was rescheduled for these patients. Additionally, another child whose caregiver tested positive had surgery rescheduled despite the child testing negative for SARS-CoV-2 (Table 3). The median delay in surgery was 25 days. There was no specific surgical morbidity in these patients with COVID-19. Postoperatively, 26 children were tested because of specific symptoms or contact history from the community, and 6 (23%) tested positive for SARS-CoV-2 between postoperative days 14 to 44 (Table 4). The infected patients in the postoperative period included a child who got reinfected after a prior infection in the preoperative period. The child following surgery tested positive again on the 23rd postoperative day before receiving adjuvant radiotherapy. Since all these 6 children had a negative preoperative test, they were outside the incubation period for SARS-CoV-2. The surgical procedures performed in these patients included a cavity surgery in 5 patients (nephrectomy in 2, abdominal neuroblastoma, retrovesical sarcoma, chest wall Ewing's sarcoma excision in 1 patient each) and a trunk soft tissue sarcoma excision along with intraoperative brachytherapy in 1. The caregiver of an infant became positive on the first postoperative day while the child was negative for SARS-CoV-2. Due to this, both the mother and child were placed in isolation. None of the postoperative patients with COVID-19 required ICU admission; however, they endured a delay in initiating adjuvant treatment (median delay = 22 days). An autologous stem cell transplant was deferred in a child with high-risk neuroblastoma. The child developed brain metastasis in 2.5 months from the surgery.

**Table 3:** Details of the patients/caregivers with SARS-CoV-2 infection in the preoperative period.

Age, yr	Sex	Diagnosis	Reason for testing	Surgery	Delay in surgery (d)	Treatment for COVID	CD Grade	Status at last follow-up (days since positive test)
4	M	Biliary Rhabdomyosarcoma	Preoperative	Pancreatoduodenectomy with right hepatectomy	28	Symptomatic	III^∗^	Stable on adjuvant chemotherapy (98)
4	M	Undifferentiated sarcoma- pelvis	Preoperative	Retrovesical sarcoma excision	25	Symptomatic	0	Reinfection. Completed treatment. (186)
5	M	Wilms tumor with intra-atrial thrombus	Preoperative (caregiver positive, child negative)	Nephrectomy with intra-atrial thrombectomy under cardiopulmonary bypass.	16	Symptomatic	0	Stable on adjuvant chemotherapy (136)

CD = Clavien Dindo score, SARS-CoV-2 = severe acute respiratory syndrome coronavirus 2.

∗ CD GRADE III – In view of drainage of intra-abdominal collection under general anesthesia.

**Table 4:** Details of the patients/caregivers with SARS-CoV-2 infection in the postoperative period.

Age, yr	Sex	Diagnosis	Surgery	Reason for testing	Postoperative day positive	Delay in adjuvant therapy, days	Treatment for COVID	Status at last follow-up (days since positive test)
4	M	Undifferentiated Sarcoma Pelvis	Retrovesical Sarcoma Excision	Fever	23	35	Symptomatic	Stable, completed treatment. (186)
3	M	Rhabdomyosarcoma	Excision with Brachytherapy	Fever	32	21	Symptomatic	Stable, on adjuvant chemotherapy (180)
3	M	Wilms Tumor	Nephrectomy	Fever	33	15	Symptomatic	Stable, completed treatment. (180)
2	F	Wilms Tumor	Nephrectomy with IVC thrombectomy	Fever	25	30	Symptomatic	Stable, on adjuvant chemotherapy (90)
4	F	Neuroblastoma	Abdominal Neuroblastoma Excision	Fever	14	ASCT Deferred	Symptomatic	Brain Metastases (150)
14	M	Ewing's sarcoma ribs	Rib excision with lobectomy and vascular graft	Fever	44	22	Symptomatic	Stable, on adjuvant chemotherapy (90)
0.4	M	Mature teratoma maxilla	Total Maxillectomy	(caregiver symptomatic	1	-	Symptomatic	Stable (150)

ASCT = autologous stem cell therapy, SARS-CoV-2 = severe acute respiratory syndrome coronavirus 2.

When compared to the previous year of 2019 (March to November), there was a 43% reduction in the number of cases operated (220 versus 125) and a 47.4% reduction in the new case registration (1651 versus 868) in the Department of Pediatric Oncology. During this period, no surgical residents or faculty acquired COVID-19; however, 4 residents in pediatric medical oncology and 4 support staff tested positive. None of these personnel developed severe symptoms or required hospital or ICU care.

## 4 Discussion

The results of this study reflect the safety of continuing elective cancer surgery in children during these challenging times. A previous study from our center in the earlier period of the pandemic had also confirmed this for all cancers.^[3]^ The surgeries performed in the study period included a comprehensive range of tumor surgeries, including nephrectomies, neuroblastoma, liver, and soft tissue tumor resections. Even complex surgeries like extended liver resections, Whipple's procedure with hepatectomy, intra-atrial thrombectomy under cardiopulmonary bypass, and free microvascular flaps were performed. There are about 1 million new cancer cases in India every year, of which around 0.2 million will require surgery.^[3,9]^ Childhood cancers account for about 1 to 4.9% of this burden.^[10]^ The cancer mortality in the absence of definitive surgery where indicated will considerably exceed the mortality due to COVID-19. These observations were compelling enough to continue elective major cancer surgeries at our institute.^[1–3]^

A total of 7 children with COVID-19, including reinfection, occurred in this cohort of patients. A low infection rate of 2% was observed in the asymptomatic preoperative patients, although 18% were not tested. A similar infection rate in asymptomatic children with cancer has been reported from the Memorial Sloan Kettering Cancer Centre, New York (2.5%), and Lombardia, Italy (3.2%).^[5,6]^ The infection rates in the symptomatic postoperative patient were 23%, compared with the 29.3% positivity rates in the symptomatic patients at Memorial Sloan Kettering Cancer Centre. It is unlikely that the 6 children with postoperative infection were in the incubation period just prior to surgery as the minimum time to first positivity was 14 days. Moreover, they are less likely to have contracted COVID during surgery and recovery in ICU as SOPs are strictly maintained in the care of patients with COVID-19 and without COVID-19 and in conducting elective and emergency surgeries. The most likely time of infection seems to be the postoperative period. They could have contracted the infection from the ward (most likely from non-healthcare staff such as other patients or their relatives) and or at their accommodation (and the travel to visit hospital thereof) since the community spread was high during the period.

COVID-19 in children is typically associated with a mild clinical course and low asymptomatic carrier rates, even in children with cancer.^[5–7,11–16]^ The possible reason for this difference includes varying expression or distribution of angiotensin-converting enzyme-2 receptors for SARs-CoV-2; immaturity of the immune system which fails to mount an aggressive cytokine response; potential immunity from respiratory viruses which cross protects against SARs-CoV-2; maternal antibodies transferred through the placenta or breast milk; cross-immunity from vaccination with some live vaccines like the oral polio vaccine, measles vaccine or BCG.^[13–16]^ The clinical course of patients with COVID-19 in this study was also mild, and all had an uneventful recovery with symptomatic treatment only, although there was reinfection in 1 patient. The children with COVID-19 preoperatively underwent complex surgeries after recovery from the infection. There were no complications related to COVID-19 after the surgery. In the postoperative period, the patients who acquired COVID-19 received adjuvant treatment and did not endure any COVID-19 related complication.

The only adverse impact of COVID-19 was a delay in surgical or adjuvant treatment. However, this delay did not influence the short-term outcomes; its impact on the long-term oncological outcomes is uncertain. The disease progression in the child with high-risk neuroblastoma could probably be due to the aggressive disease rather than deferment of autologous stem cell transplant since the progression was within 2.5 months of the surgery.

Comparing the data from the March-to-November period of 2019 with 2020, there was a 47% decline in new cases registered and a 43% reduction in total cases operated. This decline reflects the restricted access to health care due to the imposed travel constraints, decreased regional and national referral, and caregivers’ fears to avert the risk of acquiring COVID-19. The number of undiagnosed cancer cases in this period is elusive now; however, the impact of these missed cases in the national and international statistics of childhood cancer will be significant. Another consequential issue is the presentation of patients at a more advanced stage of the disease due to the lockdown.^[17]^ This is particularly important in solid tumors where possible outcomes are affected by the initial stage of presentation. The results of this study should encourage families to seek early healthcare and avoid discontinuation of treatment. In addition, the proven efficacy of PPE in protecting against contracting the illness and preventing spread should motivate the healthcare providers to maintain SOP's, particularly as we navigate an unpredictable road ahead with the fear of a second wave of infection and the possibility of future lockdowns.

Although our results humbly represent the largest series of elective cancer surgeries for children, this study has certain limitations. The COVID-19 testing was not uniformly performed in all patients preoperatively, given the institutional policy in the early phase of the pandemic. Additionally, the testing was not performed for all caregivers and the staff associated with their treatment delivery leading to a potential bias in determining the infection status, especially asymptomatic carriers. Finally, the relatively limited number of patients with COVID-19 in this cohort limits the generalization of results to all children with cancer; however, the low infection rate compares with the infection rates at few other centers treating children with cancer.

## 5 Conclusion

COVID-19 should not be a deterrent for continued cancer care, especially surgical intervention. Cancer surgeries, including complex surgical procedures, can be safely performed by adopting universal preventive measures. Likewise, caregivers and centers dealing with childhood cancers can be assured of continued cancer care without any added morbidity from COVID-19. A concerted effort amongst all service providers and a robust organizational superintendence remain the key to achieving this goal.

## Author contributions

**Conceptualization:** Sajid S. Qureshi.

**Data curation:** Sajid S. Qureshi, Deepak Ramraj.

**Formal analysis:** Sajid S. Qureshi, Deepak Ramraj.

**Investigation:** Sajid S. Qureshi, Deepak Ramraj.

**Methodology:** Sajid S. Qureshi, Deepak Ramraj.

**Project administration:** Sajid S. Qureshi.

**Resources:** Sajid S. Qureshi.

**Software:** Sajid S. Qureshi.

**Supervision:** Sajid S. Qureshi, Girish Chinnaswamy, Badira C Parambil, Maya Prasad, Nayana Amin, Subramaniam Ramanathan, Nehal Khanna, Siddharth Laskar.

**Validation:** Sajid S. Qureshi, Siddharth Laskar.

**Visualization:** Sajid S. Qureshi.

**Writing – original draft:** Sajid S. Qureshi, Deepak Ramraj, Girish Chinnaswamy, Badira C Parambil, Maya Prasad, Nayana Amin, Subramaniam Ramanathan, Nehal Khanna.

**Writing – review & editing:** Sajid S. Qureshi, Girish Chinnaswamy, Badira C Parambil, Maya Prasad, Nayana Amin, Subramaniam Ramanathan, Nehal Khanna, Siddharth Laskar.
